# Long-Term Posttraumatic Growth in Victims of Terrorism in Spain

**DOI:** 10.3389/fpsyg.2022.847099

**Published:** 2022-03-23

**Authors:** Rocío Fausor, Jesús Sanz, Ashley Navarro-McCarthy, Clara Gesteira, Noelia Morán, Beatriz Cobos-Redondo, Pedro Altungy, José M. S. Marqueses, Ana Sanz-García, María P. García-Vera

**Affiliations:** Departamento de Personalidad, Evaluación y Psicología Clínica, Facultad de Psicología, Universidad Complutense de Madrid, Madrid, Spain

**Keywords:** posttraumatic growth, PTSD, psychological consequences, terrorism, victims, depression, optimism

## Abstract

**Background:**

Scientific literature on posttraumatic growth (PTG) after terrorist attacks has primarily focused on persons who had not been directly exposed to terrorist attacks or persons who had been directly exposed to them, but who were assessed few months or years after the attacks.

**Methods:**

We examined long-term PTG in 210 adults directly exposed to terrorist attacks in Spain a mean of 29.6 years after the attacks (range: 2–47 years). The participants had been injured by a terrorist attack (38.6%) or were first-degree relatives of people who had been killed or injured by a terrorist attack (41.4% and 20%, respectively). They completed diagnostic measures of emotional disorders and measures of PTSD and depression symptomatology, optimism, and PTG.

**Results:**

Multiple regression analyses revealed gender differences (women reported higher levels of PTG than did men) and a positive linear relationship between PTG and cumulative trauma after the terrorist attack. Some PTG dimensions were significantly associated with PTSD symptomatology, these associations being linear, not curvilinear. However, PTG was not associated with depression symptomatology, diagnosis of emotional disorders, age, elapsed time since the attack, or optimism. In comparison with survivors assessed 18 years after the 1995 Oklahoma City bombing, Spanish victims of terrorism showed higher levels of appreciation of life, but lower levels of relating to others and spiritual change.

**Conclusion:**

The findings underscore the influence of gender on PTG and provide support to the hypothesis that some emotional distress may be a necessary condition of PTG. Future studies on PTG after terrorist attacks should take into consideration the characteristics of the terrorist attack itself and the contexts of violence and threat in which it occurred. The political, social, and cultural characteristics of the community affected by it and the profile and characteristics of other traumatic events suffered after the attack should also be taken into account in further research.

## Introduction

The scientific literature has revealed that a significant percentage of the people who have suffered a terrorist attack can show different psychological disorders and symptoms because of it. In particular, victims can develop posttraumatic stress disorder (PTSD) or major depressive disorder (MDD). These mental health problems can be exhibited short, medium, and long term after the terrorist attack (García-Vera et al., 2021; Sanz and García-Vera, 2021). This can be seen in the systematic review conducted by García-Vera et al. (2016), where a year after suffering a terrorist attack, the prevalence of PTSD among direct adult victims was 33–39%. Among family members of the deceased or those who had been injured in the attacks, the prevalence was between 17 and 29%, and 4% among the general population belonging to the area that was affected by the attack. In the systematic review conducted by Salguero et al. (2011), it was estimated that the prevalence of MDD among direct adult victims was between 20 and 30%, and of 4–10% among the general population belonging to the area that was affected by the attack.

Nevertheless, as it can be inferred by the previously presented percentages, not all people exposed to a terrorist attack will come to show mental disorders or symptoms. In fact, it is estimated that a high percentage of direct victims, between 60 and 70%, will not develop any kind of mental disorder months after suffering the attack (García-Vera et al., 2021; Sanz and García-Vera, 2021).

Furthermore, a growing scientific literature has proven that, after a terrorist attack, people can experience positive reactions. For example, people can develop strengths and new abilities, increase their altruistic behaviors, strengthen the presence of positive emotions, and show positive changes in cognitive schemas about humankind, the world or oneself (Vázquez et al., 2008). These positive reactions are encompassed in the construct of posttraumatic growth (PTG), which has been defined as “positive psychological change experienced as a result of the struggle with highly challenging life circumstances” (Tedeschi and Calhoun, 2004, p. 1). This construct has mainly been assessed by the Posttraumatic Growth Inventory (PTGI; Tedeschi and Calhoun, 1996). The PTGI provides a global score of posttraumatic growth, as well as scores of five of its different components or dimensions: Relating to Others, New Possibilities, Personal Strength, Spiritual Change, and New Appreciation of Life. In fact, both the full version and the short form of the PTGI, the PTGI-SF (Cann et al., 2010), are the most used assessment tools for PTG. These assessment tools are used in studies included in various meta-analyses, such as the ones published by Vishnevsky et al. (2010), Liu et al. (2017), and Wu et al. (2019).

Studies included in the meta-analyses mentioned above have examined that PTG can appear after suffering different types of traumatic events, such as natural disasters, traffic accidents, severe health conditions, and sexual or physical assault. However, hardly any of these studies address that PTG can develop after suffering a terrorist attack. To be precise, none of the 63 studies in the meta-analysis conducted by Liu et al. (2017), or the 26 studies in the meta-analysis conducted by Wu et al. (2019), examined the appearance of PTG after a terrorist attack. Out of the 70 studies included in the Vishnevsky et al. (2010) meta-analysis, only five cover this topic. In fact, a search was conducted on December 14, 2021, using the bibliographic database PsycInfo, with the words “posttraumatic growth” or “post-traumatic growth” and “terrorist” or “terrorism.” These words were searched for in the document title or abstract fields. The search revealed 51 publications, out of which only 26 reported an empirical research study on PTG after a terrorist attack (excluding three that were qualitative studies or case studies and did not offer quantitative data on PTG).

Seventy-seven percent of the 26 studies were either (1) focused on people who had not been directly exposed to a terrorist attack, meaning they had not been injured, nor did they know people who had been wounded or killed by it (e.g., Butler et al., 2005; Páez et al., 2007; Park et al., 2008, 2012; Rimé et al., 2010), or (2) focused on people who had been directly exposed to a terrorist attack but had been evaluated a few months after the attack, or 1 or 2 years after at most (e.g., Blix et al., 2016; Ikizer and Ozel, 2021). However, there are not many studies that examine PTG in victims of terrorism who have been evaluated many years after being directly exposed to the attack. In fact, only six studies were found in the search that was mentioned in the previous paragraph, and four of them were based on the same two samples of survivors (Tucker et al., 2016, 2018; Spano, 2018; Cárdenas Castro et al., 2019; Glad et al., 2019, 2020).

The research of PTG in that type of population is essential, given that different studies have found that a greater level of posttraumatic stress symptoms is associated with a greater level of PTG (see the meta-analysis of Liu et al., 2017). In this sense, given that people directly exposed to a terrorist attack show a greater prevalence of PTSD than people indirectly affected by it (García-Vera et al., 2016, 2021; Sanz and García-Vera, 2021), it is presumed that people directly exposed to a terrorist attack will show very high levels of PTG. In contrast, some studies have found that the relationship between posttraumatic stress symptoms and PTG is not linear, but curvilinear. Specifically, the relationship has the shape of an inverted U, meaning highest levels of PTG would be associated with moderate levels of posttraumatic stress symptoms, not with high or low levels of posttraumatic stress symptoms (see the meta-analysis of Shakespeare-Finch and Lurie-Beck, 2014). Consequently, the matter is not settled yet and requires further research. It would be beneficial to conduct more studies examining the presence of PTG in people who have been directly exposed to a terrorist attack, as this population shows greater variability in posttraumatic stress symptoms. Therefore, these kinds of studies would help examine more fully the relationship between PTG and posttraumatic stress symptoms.

Previous studies have also found a relationship between the level of PTG and the elapsed time since the traumatic event (Linley and Joseph, 2004; Wu et al., 2019). For instance, the meta-analysis conducted by Wu et al. (2019) found that the presence of moderate levels of PTG was associated with the elapsed time since the traumatic event. This meta-analysis showed that a shorter elapsed time since the traumatic event (less than 6 months) was associated with a greater degree of moderate-high PTG. That being said, most studies (60%) included in the meta-analysis conducted by Wu et al. (2019) assessed the presence of PTG between a day and 24 months after the traumatic event. Only five studies (16.7%) assessed the presence of PTG after six or more years since the occurrence of the traumatic event. For this reason, it might be possible to find a curvilinear relationship when examining longer periods of time. In particular, the relationship between elapsed time and PTG could be similar to the inverted U shape found in studies on posttraumatic stress symptomatology and PTG.

There is still no agreement on what is understood by long-term PTG. In the context of people who have had cancer, Lelorain et al. (2010) consider that long-term PTG is that which appears 5 years after being diagnosed with cancer. This time limit seems a bit arbitrary, but taking it as a starting point, several studies in the field of cancer have shown the existence of long-term PTG after 5–8 years of cancer diagnosis (Lechner et al., 2006), after 10 years (Mols et al., 2009), or after 5–15 years (Lelorain et al., 2010). Concerning other traumatic events, the existence of long-term PTG has been proven even after longer periods, for example, 11–24 years after the Vietnam War (Fontana and Rosenheck, 1998) or even 52 years after the Dresden bombing during World War II (Maercker and Herrle, 2003). However, as the meta-analysis by Wu et al. (2019) has shown, most research has examined PTG in the short to medium term, that is, within the first 5 years since the traumatic event.

Out of the six studies on long-term PTG in people directly exposed to a terrorist attack, two studies examined PTG in teenagers or young people (Glad et al., 2019, 2020), one study examined PTG in a very small sample of adults (*n* = 7; Spano, 2018), and one study examined PTG in adults exposed to a very specific type of terrorism such as state terrorism (Cárdenas Castro et al., 2019). Therefore, only the two studies conducted by Tucker et al. (2016, 2018) seem relevant for the purposes of the present study. Both studies, as well as the present one, have examined long-term PTG in a sufficiently large sample of adults who have been directly exposed to a similar type of terrorism.

Tucker et al. (2016, 2018) used the PTGI-SF to assess the PTG level of 138 terrorism survivors 18.5 years after the 1995 Oklahoma City bombing. The results showed a relationship of PTG with the variables sex, education level, and posttraumatic stress symptoms, but not with the variables age, marital status, or depressive symptoms. In particular, a higher level of posttraumatic stress symptoms, being a woman or being a graduate student was associated with a higher PTG. The results also indicated that many years later, more than 30% of the survivors showed high or very high levels of PTG in seven of the 10 items that compose the PTGI-SF. In fact, averaging the percentages obtained in the 10 items, it could be estimated that 32.3% of the survivors showed high or very high levels of PTG, and 55% showed moderate, high, or very high levels.

Showing moderate, high, or very high levels of PTG in any item of the PTGI or the PTG-SF means that the score is equal to or greater than 60% of the highest possible score of the item. This criterion, applied to individual items or to the total score of any other PTG measurement instrument, was the one that Wu et al. (2019) used in their meta-analysis on the prevalence of moderate to high levels of PTG after any type of traumatic event. After reviewing 26 studies that evaluated a total of 10,181 people, Wu et al. (2019) found that the prevalence of moderate-high PTG ranged between 10% and 77.3%, with a mean prevalence of 52.6%. This percentage is similar to that found by Tucker et al. (2016) in survivors 18.5 years after suffering a terrorist attack (55%). This suggests that, even after directly suffering a traumatic event as devastating as a terrorist attack and even after many years since the attack, a significant percentage of people report PTG.

The general objective of the present study was to extend the scarce existing research on PTG in people who have been directly exposed to a terrorist attack a long time ago. To do so, we assessed PTG in a large sample of survivors and close relatives of people who had been killed or injured in terrorist attacks in Spain. The assessment took place between 2 and 47 years after having suffered the attack. Spain has a long history of terrorist attacks, starting in the 60s of the 20th century. Since this date, at least 1,431 people have lost their lives at the hands of various terrorist organizations. Most of these deaths —about 857— and most of the terrorist attacks were caused by a terrorist organization called ETA. ETA’s terrorist activity lasted for 50 years, between 1961 and 2011, although it was particularly intense between the late 1970s and early 1990s. It is in 2011 when ETA announced the definite cessation of its armed activity (Sanz and García-Vera, 2021).

The specific objectives of the present study were to: (1) examine the prevalence of long-term PTG in people directly exposed to a terrorist attack; (2) compare this prevalence with that found in the only previous studies that have examined long-term PTG in a similar sample of participants, that is, the studies by Tucker et al. (2016, 2018); (3) examine the linear and curvilinear relationship between PTG and posttraumatic stress, and between PTG and the elapsed time since the terrorist attack; and (4) test the associations between PTG and other variables that the scientific literature has already proven to be linked with, such as the sex of the person (Vishnevsky et al., 2010), depressive symptoms (Long et al., 2021), and optimism (Prati and Pietrantoni, 2009).

## Materials and Methods

### Participants

A sample of 210 adults was recruited to carry out the present study. They had all been directly exposed to a terrorist attack and were members of the Association of Victims of Terrorism (AVT) of Spain. They were also part of a larger study on the long-term psychological consequences of terrorist attacks. The selection of this sample of participants was carried out in two phases. In the first phase, 791 adults belonging to the AVT were contacted by telephone. Out of them, 390 completed a psychological interview by telephone, while 38 participants requested to be interviewed in person. In a second phase, the 428 victims interviewed in the first phase were invited to undergo a more comprehensive face-to-face psychological assessment. This assessment included various psychopathological questionnaires, including the PTGI, and a structured diagnostic interview for emotional disorders. Out of the 428 people invited, 210 performed this second face-to-face psychological evaluation.

The average age of this final sample of participants was 53.48 years (range = 18–84; *SD* = 12.78) and 49.5% of them were women. Out of these participants, 38.6% had been injured in a terrorist attack, 41.4% were close relatives of a person who had been killed in a terrorist attack, and the remaining 20% were close relatives of a person who had been injured in a terrorist attack. The terrorist attacks suffered by the participants had occurred an average of 29.61 (range = 2–47; *SD* = 11.02) years before they participated in this study, and most of them had been carried out by ETA (89.5%). Concerning education, 44.2% of the participants had secondary education, 31.3% had university studies, 23.5% had primary education, and 1% had no formal studies. The majority of participants (66.2%) were married or living with a stable partner and were working at the time of assessment (55.7%). The results of the diagnostic interview indicated that 46.55% of the participants suffered from an emotional disorder, the most frequent being MDD (17.6%), PTSD (16.3%), specific phobia (8.2%), panic disorder (8.1%), and generalized anxiety disorder (7.1%).

### Instruments

*Structured Clinical Interview for Axis I Disorders of the DSM-IV, Clinician Version* (SCID-I CV; First et al., 1997; Spanish version in First et al., 1999). The SCID-I CV evaluates the presence of diagnosable mental disorders according to the DSM-IV. In the present research, only modules A (affective episodes) and F (anxiety and other disorders) were applied for the diagnosis of PTSD, MDD, or other emotional disorders (e.g., anxiety disorders). The diagnostic measures of SCID-I CV have good psychometric properties, including good inter-rater reliability and test–retest indices for the diagnosis of both PTSD and MDD (Zanarini et al., 2000; Lobbestael et al., 2011).

*Beck Depression Inventory-II* (BDI-II; Beck et al., 1996; Spanish adaptation in Beck et al., 2011). The BDI-II is a self-report instrument designed to assess the presence of depressive symptoms and their severity. It consists of 21 items that are scored from 0 to 3, offering a range of scores from 0 to 63. The BDI-II presents, both in its original version and in its Spanish adaptation, good indices of reliability and validity (Beck et al., 1996, 2011; Sanz et al., 2005). In the terrorism victim sample of the present study, the BDI-II obtained an internal consistency index (alpha) of 0.95.

*PTSD Checklist for DSM-5* (PCL-5; Weathers et al., 2013; Spanish adaptation in Sanz et al., under review^1^). The PCL-5 consists of 20 items that evaluate symptoms of PTSD according to the DSM-5. Each item is answered with five-point Likert scales and scored from 0 to 4, offering a range of scores from 0 to 80. Both the original version of the PCL-S and its Spanish adaptation have good indices of reliability, convergent validity, and diagnostic validity (Blevins et al., 2015; see text footnote 1). Participants in this study were asked to complete the PCL-5 bearing in mind their experience with the terrorist attack they had suffered. Their scores on the PCL-5 obtained an internal consistency index (alpha) of 0.95.

*Life Orientation Test-Revised* (LOT-R; Scheier et al., 1994; Spanish adaptation in Otero et al., 1998). The LOT-R is an instrument designed to measure dispositional optimism. It consists of 10 items, answered with five-point Likert scales and with its scores ranging from 0 to 4. A total score of dispositional optimism can be obtained from six out of the 10 items and can range between 0 and 24. Both the original version of the LOT-R and the Spanish adaptation have good indices of reliability and validity (Scheier et al., 1994; Ferrando et al., 2002). In the terrorism victim sample of the present study, the LOT-R obtained an internal consistency index (alpha) of 0.80.

*Posttraumatic Growth Inventory* (PTGI; Tedeschi and Calhoun, 1996). The present study used the Spanish adaptation of Vázquez et al. (2006). The instructions of this adaptation specifically ask respondents to indicate their “reaction after the terrorist attack.” The PTGI is an instrument designed to assess positive psychological changes that can be experimented after a traumatic or highly stressful life event. The PTGI consists of 21 items that are answered with six-point Likert scales. These scales range from “I did not experience this change” (scored 0) to “I experienced this change to a very great degree” (scored 5). This instrument offers a global score of PTG that can range between 0 and 105. Based on the results of various factor analyses (Tedeschi and Calhoun, 1996; Taku et al., 2008), the PTGI can also be used to measure five different dimensions of PTG through the following five subscales: Relating to Others, Appreciation of Life, New Possibilities, Spiritual Change, and Personal Strength. The total scores, as well as the scores of these subscales, show good indices of reliability and validity (Tedeschi and Calhoun, 1996; Taku et al., 2008). In the terrorism victim sample of the present study, the total scores of the PTGI obtained an internal consistency index (alpha) of 0.95, while the scores of the PTGI subscales obtained the following indices: 0.82 (Relating to Others), 0.89 (New Possibilities), 0.84 (Personal Strength), 0.74 (Appreciation of Life), and 0.81 (Spiritual Change). The total score of the 10 items that constitute the PTGI-SF (Cann et al., 2010) was also calculated, since this version was used in the studies of Tucker et al. (2016, 2018). The PTGI-SF total score can range between 0 and 50. In the terrorism victim sample of the present study, the total scores of the PTGI-SF showed an internal consistency index (alpha) of 0.89.

### Procedure

Every participant’s verbal informed consent was obtained prior to the telephone interview. During the face-to-face interview, they also signed an informed consent form to take part in a larger study on the long-term psychological consequences of terrorism. Subsequently, a psychologist assessed the psychological consequences derived from the attack or attacks. To do so, the following instruments were administered in this order: SCID-I VC, BDI-II, PCL-S, PTGI, and LOT-R. All psychologists who acted as evaluators had been specifically trained in conducting the assessments through a university diploma focused on psychological care for victims of terrorist attacks. They were also trained by observing assessments, conducting supervised assessments, and conducting weekly clinical sessions.

### Data Analysis

Statistical analyses were carried out with SPSS, version 25, and with the online calculators of SciStat.com (MedCalc Software Ltd., Ostend, Belgium). The mean of the PTGI and PTGI-SF scores were calculated, as well as the prevalence of moderate to very high levels of PTG. These means and prevalence were compared with the results obtained in previous studies (Tucker et al., 2016, 2018; Wu et al., 2019) with Student’s *t*-tests for independent samples and with chi-square tests for independent samples, respectively.

To analyze the factors that are related to the presence of long-term PTG, the Pearson correlations of the PTG measures with the measures of the following sociodemographic, clinical, and attack-related characteristics were calculated: sex (1 = women; 0 = men), age, level of education (without studies, primary, secondary and university studies), marital status (married or living as a couple vs. single, divorced, separated or widowed), posttraumatic stress symptoms, depressive symptoms, PTSD diagnosis, MDD diagnosis, anxiety disorder diagnosis, absence of diagnosed emotional disorders, elapsed years since the attack, age at the time of the attack, number of attacks suffered, number of traumatic events suffered after the terrorist attack, having been injured in the attack (vs. relative of the deceased or injured in the attack), and being a relative of the deceased in the attack (vs. injured in the attack or relative of the injured in the attack).

Subsequently, multiple regression analyses were performed on each of the measures of PTG using as predictors the variables that would have shown a statistically significant correlation (*p* < 0.05) in the present study. As a prior step before the regression analyses, the possible existence of collinearity problems among the predictors was tested by calculating the tolerance rates and variance inflation factors (VIF). It was taken into account that tolerance rates below 0.20 are indicative of potential collinearity problems, and that indices below 0.10 indicate serious problems. Furthermore, VIFs above 12 also suggest a problem of collinearity (Martínez Arias et al., 2015).

To examine the presence of inverted U-shaped curvilinear relationships between PTG and the sociodemographic, clinical, and attack-related continuous variables, the scores on these variables were first mean-centered (subtracting the mean from each score) and then squared to create the quadratic term for those variables. Hierarchical regression analyses were performed for each PTG measure. In each analysis, the linear effect or term of each variable (the mean-centered variable) was included in Step 1, and the quadratic effect or term (the squared mean-centered variable) in Step 2. Thus, it was examined whether the inclusion of the quadratic term implied a statistically significant increase in the explained variance of the PTG measure.

## Results

### Posttraumatic Growth in Victims of Terrorist Attacks

The mean total PTGI score for the sample of participants was 41.78 (*SD* = 27.32), indicating that, on average, the participants reported experiencing positive psychological changes of mild intensity. The presence of moderate-high PTG can be defined with the criterion of a score equal to or greater than 60% of the maximum score (Wu et al., 2019), which translates into a score equal to or higher than 63 in the PTGI. Based on this criterion, 25.2% of the sample of victims of terrorism that took part in the present study reported moderate-high levels of PTG. This percentage was significantly lower than that found by Wu et al. (2019) in their meta-analysis on the prevalence of moderate-high PTG in victims of all types of traumatic events (25.2% vs. 52.6%; χ^2^ = 61.88, *p* < 0.0001).

Even more relevant is the comparison with the results obtained by Tucker et al. (2016) with survivors of the Oklahoma City terrorist attack 18.5 years after the attack. These survivors completed the PTGI-SF. When averaging the percentage of participants in the present study who reported experiencing moderate, high or very high positive psychological changes in every one of the 10 PTGI-SF items, 44% of the participants showed moderate-high levels of PTG. This percentage was also significantly smaller than that estimated from the study by Tucker et al. (2016) (44% vs. 55%; χ^2^ = 4.03, *p* < 0.045). Consistently, the mean total score on the PTGI-SF of the participant sample (20.53; *SD* = 13.08) was significantly lower than that obtained by Tucker et al. (2018) in the survivors of the Oklahoma City terrorist attack [20.53 vs. 23.98, *t*(346) = 2.45, *p* < 0.015], with the difference being considered small (Cohen’s *d* = 0.27).

Tucker et al. (2016, 2018) obtained a mean score of the sample of survivors of the Oklahoma City terrorist attack in each of the PTGI-SF items. They also obtained the percentage of survivors who in each item answered having experienced great or very great positive psychological changes. Table 1 shows these mean scores and percentages, comparing them with those obtained with the sample of victims of terrorism in the present study. The results of the *t*-tests for the comparison of means indicated that, in the two PTGI-SF items that measure spiritual changes, the victims of terrorism in the present study showed significantly lower mean scores, with differences being considered between small and moderate (Cohen’s *d* = 0.39 and 0.49). Furthermore, the results of the chi-square tests for the comparison of percentages indicated that a significantly lower percentage of people in the present study had experienced great or very great changes in one of these two items (“I have a stronger religious faith”), (17.9 vs. 31.2; see Table 1). The victims of terrorism in this study also showed significantly lower mean scores in one of the PTGI-SF items that measure changes in personal strength and in one of the PTGI-SF items that measure changes in relating to others, with small and moderate differences, respectively (Cohen’s *d* = 0.34 and 0.59) (see Table 1). In addition, in that last item (“I learned a great deal about how wonderful people are”), the sample of participants in this study also had a significantly lower percentage of people who had experienced great or very great changes, almost half compared to the percentage found in the sample of survivors of Tucker et al. (2016) (23.1 vs. 40.6) (see Table 1). In the remaining six items of the PTGI-SF, no statistically significant differences were found between the mean scores or between the percentages, except in one of the items. This item measures changes in the appreciation of life (“I changed my priorities about what is important in life”), and in this case, the percentage of participants in this study who showed great or very great changes was significantly higher than that found among the survivors of Tucker et al. (2016) (50.5% vs. 34.1%; see Table 1).

**TABLE 1:** Mean scores and percentages of “great/very great degree” responses in the PTGI-SF items: Oklahoma City bombing survivors (*N* = 138; Tucker et al., 2016) vs. victims of terrorism in Spain (*N* = 210).

	Oklahoma City			Victims of terrorism					
PGTI-SF items	bombing survivors			in Spain			Mean differences		Chi-square test for proportion differences
	Mean	*SD*	% of great/very great degree	Mean	*SD*	% of great/very great degree	Student’s *t*-test	Cohen’s *d*	
**Appreciation of life**									
1. I changed my priorities about what is important in life.	2.7	1.6	34.1	2.83	1.93	50.5	0.657	−0.07	9.064*
2. I have a greater appreciation for the value of my own life.	2.8	1.7	38.4	2.66	1.88	46.4	−0.706	0.08	2.165
**New possibilities**									
3. I am able to do better things with my life.	2.2	1.7	24.6	1.78	1.77	22.5	−2.199	0.24	0.205
6. I established a new path for my life.	1.8	1.8	26.1	2.03	1.89	30.0	1.132	0.12	0.620
**Spiritual change**									
4. I have a better understanding of spiritual matters.	2.4	1.9	34.1	1.65	1.91	24.8	−3.591*	0.39	3.525
8. I have a stronger religious faith.	2.2	1.9	31.2	1.29	1.79	17.9	−4.527*	0.49	8.250*
**Relating to others**									
5. I have a greater sense of closeness with others.	2.2	1.7	23.2	1.71	1.83	23.5	−2.513	0.28	0.004
10. I learned a great deal about how wonderful people are.	2.8	1.6	40.6	1.80	1.80	23.1	−5.295*	0.59	12.100*
**New possibilities**									
7. I know better that I can handle difficulties.	2.7	1.6	34.1	2.11	1.84	28.7	−3.079*	0.34	1.135
9. I discovered that I’m stronger than I thought I was.	2.4	1.8	36.2	2.62	1.96	44.0	1.058	−0.11	2.090

*Statistically significant at p < 0.05 level with Bonferroni correction (p < 0.005 without Bonferroni correction).

The sample of participants included in the study conducted by Tucker et al. (2016) and the sample included in the present study differ in the time elapsed since the terrorist attack. The time elapsed in the present study ranged between 2 and 47 years. For this reason, the previous comparison analyses between both studies were repeated, but this time, using a subsample formed by participants that had suffered the terrorist attack between 10 and 25 years (both included) before taking part in this study (*n* = 47). This subsample’s time span is more similar to the one found in the study conducted by Tucker et al. (2016), where a mean of 18.5 years had elapsed since the Oklahoma City terrorist attack. The results pattern obtained with the subsample was similar to the one found with the complete sample, but given that the size of the subsample is much smaller, some differences were not statistically significant. Taking this into account, within the subsample of 47 participants, 48.93% of them showed moderate-high levels of PTG. This percentage was smaller than the one obtained in the study carried out by Tucker et al. (2016), although the difference was not statistically significant (48.9% vs. 55%; χ^2^ = 0.52, *p* = 0.474). Consistently, the mean total PTGI-SF score found in the subsample of 47 participants (22.04; *SD* = 10.90) was smaller than the one obtained by Tucker et al. (2016, 2018), but the difference was not statistically significant [22.04 vs. 23.98; *t*(183) = 0.95, *p* = 0.345; Cohen’s *d* = 0.16]. Nevertheless, there were statistically significant and moderate differences in one of the two PTGI-SF items that assess spiritual changes, and also in one of the items that assess changes in relating to others (Cohen’s *d* = 0.55 and 0.56, respectively; see Table 2). Furthermore, the subsample of participants also obtained a significantly smaller percentage of people who had experienced high or very high change regarding both items of the PTG-SF (respectively, 10.7% and 19.1% vs. 31.2% and 40.6%; see Table 2). This being said, these differences were not statistically significant once the Bonferroni correction was performed to control the making of multiple comparisons. Finally, the percentage of the subsample that reported high or very high changes in one of the items that assesses change in the appreciation of life was significantly higher than the one reported by the survivors in the study conducted by Tucker et al. (2016) (63.8% vs. 34.1%) (see Table 2).

**TABLE 2:** Mean scores and percentages of “great/very great degree” responses in the PTGI-SF items: Oklahoma City bombing survivors (*N* = 138; Tucker et al., 2016) vs. victims of terrorism in Spain who suffered a terrorist attack 10–25 years ago (*n* = 47).

	Oklahoma City			Victims of terrorism					
PGTI-SF items	bombing survivors			in Spain			Mean differences		Chi-square test for proportion differences
	Mean	*SD*	% of great/very great degree	Mean	*SD*	% of great/very great degree	Student’s *t*-test	Cohen’s *d*	
**Appreciation of life**									
1. I changed my priorities about what is important in life.	2.7	1.6	34.1	3.32	1.67	63.8	2.269	−0.38	12.657**
2. I have a greater appreciation for the value of my own life.	2.8	1.7	38.4	3.04	1.64	55.3	0.843	−0.14	4.071
**New possibilities**									
3. I am able to do better things with my life.	2.2	1.7	24.6	1.83	1.62	21.3	−1.304	0.22	0.210
6. I established a new path for my life.	1.8	1.8	26.1	2.43	1.85	36.2	2.058	−0.34	1.740
**Spiritual change**									
4. I have a better understanding of spiritual matters.	2.4	1.9	34.1	1.79	1.92	27.6	−1.896	0.32	0.672
8. I have a stronger religious faith.	2.2	1.9	31.2	1.21	1.67	10.7	−3.177**	0.55	7.618*
**Relating to others**									
5. I have a greater sense of closeness with others.	2.2	1.7	23.2	1.91	1.65	21.2	−1.018	0.17	0.080
10. I learned a great deal about how wonderful people are.	2.8	1.6	40.6	1.91	1.60	19.1	−3.294**	0.56	7.072*
**New possibilities**									
7. I know better that I can handle difficulties.	2.7	1.6	34.1	2.15	1.60	23.4	−2.035	0.34	1.854
9. I discovered that I’m stronger than I thought I was.	2.4	1.8	36.2	2.45	1.88	40.4	0.163	−0.03	0.263

*Statistically significant at p < 0.05 level with Bonferroni correction (p < 0.005 without Bonferroni correction).

**Statistically significant at p < 0.10 level with Bonferroni correction (p < 0.01 without Bonferroni correction).

### Variables Related to Posttraumatic Growth: Linear Relationship

Table 3 presents the linear correlations of the six measures of PTG —total scale and five subscales of PTGI— with the sociodemographic, clinical, and attack-related variables assessed in the present study. As can be seen in Table 3, the only variable that showed statistically significant correlations with all the six PTG measures was sex, with coefficients between small and almost medium that ranged between 0.17 (with spiritual change) and 0.29 (with personal strength). These coefficients indicated that women presented higher PTG than men, both globally and in each of its dimensions. The number of traumatic events a person has been exposed to after suffering a terrorist attack showed statistically significant correlations with five out of the six measures of PTG. The correlations were small, ranging between 0.17 (for new possibilities) and 0.21 (for a new appreciation of life). These coefficients indicate that an increase in the number of traumatic events a person has been exposed to was associated with a greater degree of global PTG, as well as all of its dimensions except for spiritual growth. Two variables, marital status and posttraumatic stress symptoms, showed statistically significant correlations with two of the PTG dimensions, but not with the remaining four PTG measures. Being married or living as a couple was associated with a lower level of positive changes in relating to others and personal strength, although the association was small in both cases (*r* = −0.14). On the other hand, a higher level of posttraumatic stress symptoms was associated with a higher level of appreciation of life and spiritual change. These associations were small in both cases (*r* = 0.16 and 0.13, respectively). Three variables presented statistically significant correlations with a single PTG measure: age, the absence of diagnosed emotional disorders, and the number of attacks experienced by the victim. Older people, those with a diagnosed emotional disorder, and those who had been exposed to a greater number of attacks presented higher levels, respectively, of personal strength, appreciation of life, and positive changes in relating to others, although the associations were small in size in all three cases (*r* = 0.16, −0.14, and 0.15, respectively). The remaining variables evaluated in the present study —education level, depressive symptoms, diagnosis of PTSD, diagnosis of MDD, diagnosis of anxiety disorders, number of elapsed years since the attack, age at the time of the attack, having been injured in an attack, and being a close relative of someone who had been killed in an attack— did not show any statistically significant correlation with any of the six PTG measures of the PTGI.

**TABLE 3:** Correlations of the post-traumatic growth measures of the PTGI with the sociodemographic, clinical and attack-related variables.

Variable	Posttraumatic growth measures					
	PTGI total score	Relating to others	New possibilities	Personal strength	Appreciation of life	Spiritual change
Sex (1 = women; 0 = men)	0.24***	0.21**	0.19**	0.29***	0.19**	0.17*
Age	0.10	0.08	0.06	0.16*	0.02	0.10
Education level	−0.60	−0.02	−0.03	−0.10	−0.05	−0.05
Marital status	−0.12	−0.14*	−0.09	−0.14*	−0.03	−0.12
Optimism (LOT-R)	0.04	−0.02	0.09	0.14*	−0.01	−0.03
Depressive symptoms (BDI-II)	0.03	0.05	0.001	−0.04	0.11	0.10
Posttraumatic stress symptoms (PCL-5)	0.10	0.10	0.07	−0.01	0.16*	0.13*
PTSD	−0.005	−0.02	0.03	−0.04	0.06	0.03
MDD	0.02	0.03	0.03	−0.06	0.12	0.09
Anxiety disorder	0.05	0.03	0.01	0.08	0.06	0.005
No emotional disorder	−0.05	−0.06	−0.03	0.01	−0.14*	−0.07
No. of years elapsed since the attack	−0.04	−0.05	−0.05	0.001	−0.09	0.01
Age at the time of the attack	0.10	0.09	0.06	0.13	0.06	0.06
No. of terrorist attacks	0.13	0.15*	0.07	0.11	0.08	0.03
No. of traumatic events after the attack	0.20**	0.19**	0.17*	0.18*	0.21**	0.03
Injured vs. relative	−0.06	−0.05	−0.05	−0.12	−0.04	−0.008
Relative of deceased vs. injured or relative of injured	0.004	−0.006	0.01	0.009	0.03	0.06
Relating to others	0.93***	—	—	—	—	—
New possibilities	0.92***	0.79***	—	—	—	—
Personal strength	0.88***	0.77***	0.81***	—	—	—
Appreciation of life	0.79***	0.67***	0.67***	0.62***	—	—
Spiritual change	0.68***	0.59***	0.59***	0.49***	0.45***	—

*Statistically significant correlation at p < 0.05.

**Statistically significant correlation at p < 0.01.

***Statistically significant correlation at p < 0.001.

### Multiple Regression Analyses on Posttraumatic Growth

The eight variables that significantly correlated with some or with all of the PTGI measures were included in the multiple regression analyses on PTG, and the results are shown in Table 4. These results indicated that sex was the only variable that was significantly associated with all PTGI measures after the effects of the other predictors were controlled. The number of traumatic events experienced after suffering a terrorist attack also showed a consistent association with PTG, given that there is a significant association with five out of the six measures of PTG.

**TABLE 4:** Multiple regression analyses on posttraumatic growth (PTG) measures.

PTG measure/Predictor	Beta	*t*	*p*	Partial *r*
**PTGI total score**				
*Sex*	*0.223*	*3.07*	*0.002*	*0.216*
Age	0.112	1.67	0.097	0.120
Marital status	−0.052	−0.73	0.467	−0.053
Optimism (LOT-R)	0.080	1.01	0.314	0.073
Posttraumatic stress (PCL-5)	0.150	1.64	0.102	0.118
No emotional disorder	0.044	0.49	0.624	0.035
*No. of terrorist attacks*	*0.150*	*2.17*	*0.031*	*0.155*
*No. of traumatic events after the attack*	*0.188*	*2.77*	*0.006*	*0.196*
**Relating to others**				
*Sex*	*0.186*	*2.56*	*0.011*	*0.180*
Age	0.087	1.29	0.198	0.092
Marital status	−0.082	−1.17	0.245	−0.083
Optimism (LOT-R)	−0.011	−0.14	0.892	−0.010
Posttraumatic stress (PCL-5)	0.118	1.29	0.199	0.092
No emotional disorder	0.049	0.54	0.586	0.039
*No. of terrorist attacks*	*0.188*	*2.72*	*0.007*	*0.191*
*No. of traumatic events after the attack*	*0.182*	*2.67*	*0.008*	*0.188*
**New possibilities**				
*Sex*	*0.179*	*2.42*	*0.016*	*0.171*
Age	0.081	1.18	0.239	0.084
Marital status	−0.032	−0.453	0.651	−0.032
Optimism (LOT-R)	0.146	1.81	0.072	0.128
Posttraumatic stress (PCL-5)	0.157	1.68	0.094	0.119
No emotional disorder	0.038	0.41	0.682	0.029
No. of terrorist attacks	0.083	1.19	0.236	0.085
*No. of traumatic events after the attack*	*0.153*	*2.22*	*0.028*	*0.157*
**Personal strength**				
*Sex*	*0.277*	*3.92*	*0.001*	*0.271*
*Age*	*0.178*	*2.73*	*0.007*	*0.193*
Marital status	−0.052	−0.76	0.450	−0.054
Optimism (LOT-R)	0.138	1.79	0.075	0.127
Posttraumatic stress (PCL-5)	0.063	0.71	0.481	0.051
No emotional disorder	0.046	0.53	0.597	0.038
*No. of terrorist attacks*	*0.143*	*2.13*	*0.034*	*0.152*
*No. of traumatic events after the attack*	*0.171*	*2.59*	*0.010*	*0.183*
**Appreciation of life**				
*Sex*	*0.177*	*2.44*	*0.016*	*0.171*
Age	0.035	0.53	0.599	0.038
Marital status	0.024	0.34	0.734	0.024
Optimism (LOT-R)	0.082	1.03	0.304	0.073
*Posttraumatic stress (PCL-5)*	*0.177*	*1.91*	*0.050*	*0.135*
No emotional disorder	−0.037	−0.41	0.683	−0.029
No. of terrorist attacks	0.099	1.43	0.155	0.101
*No. of traumatic events after the attack*	*0.204*	*3.00*	*0.003*	*0.209*
**Spiritual change**				
*Sex*	*0.152*	*2.04*	*0.042*	*0.144*
Age	0.109	1.57	0.118	0.111
Marital status	−0.068	−0.94	0.348	−0.067
Optimism (LOT-R)	0.039	0.48	0.633	0.034
*Posttraumatic stress (PCL-5)*	*0.197*	*2.06*	*0.041*	*0.146*
No emotional disorder	0.055	0.59	0.554	0.042
No. of terrorist attacks	0.047	0.66	0.510	0.047
No. of traumatic events after the attack	0.019	0.27	0.790	0.019

*Statistically significant predictors at p < 0.05 are displayed in italics.*

Concerning total scores in the PTGI, the regression model with the eight predictors explained 14.7% of the variance of those scores (*R*^2^ = 0.147, *F* = 4.15, *p* < 0.001), but only the sex of the victim, the number of terrorist attacks a person has experienced and the number of traumatic events a person has been exposed to were significantly associated with the total scores of PTGI. The size and sign of the beta coefficients and the partial correlations gathered in Table 4 indicated that sex was the most important variable when it came to explaining the variance in global PTG (partial *r* = 0.22), followed by the number of traumatic events a person has been exposed to (partial *r* = 0.19) and the number of terrorist attacks a victim has suffered (partial *r* = 0.15). Taking this into account, being a woman, having suffered more traumatic events since the terrorist attack, and having lived more terrorist attacks, in this order, were associated with a higher degree of global PTG.

The complete regression model explained 13.2% of the variance of the scores in the PTGI subscale of relating to others (*R*^2^ = 0.132, *F* = 3.72, *p* < 0.001), and, once again, the variables sex, number of terrorist attacks, and number of traumatic events were significantly associated with said scores. The number of terrorist attacks a person has experienced (partial *r* = 0.19) was the most important variable when explaining the variance in positive changes when relating to others. This variable was followed, in order, by the number of traumatic events a person has suffered (partial *r* = 0.188) and his/her sex (partial *r* = 0.18). This means that experiencing a greater number of traumatic events, suffering a greater number of terrorist attacks, and being a woman were more strongly associated with the PTG dimension of relating to others.

Regarding the PTG dimension of new possibilities, the complete regression model significantly explained 10.1% of the variance of the scores of said dimension (*R*^2^ = 0.101, *F* = 2.75, *p* < 0.007), but only sex and the number of terrorist attacks a person has suffered after the terrorist attack were significantly associated with said scores (see Table 4). Sex was the most relevant variable when explaining the variance of the dimension of new possibilities (partial *r* = 0.17), followed by the number of traumatic events one has experienced (partial *r* = 0.16). This means that being a woman and having suffered a greater number of traumatic events after the terrorist attack, in this order, were associated with a higher degree to the dimension of new possibilities.

Concerning the PTG dimension of personal strength, the complete regression model significantly explained 18.7% of the variance of the scores of said dimension (*R*^2^ = 0.187, *F* = 5.58, *p* < 0.001). Sex, age, the number of traumatic events a person has experienced after the terrorist attack, and the number of terrorist attacks one has been exposed to, were significantly associated with personal strength (partial *r* = 0.27, 0.19, 0.18, and 0.15, respectively). This implies that being a woman, being older, having suffered more traumatic events after the terrorist attack, and having experienced more terrorist attacks, in this order, were associated with a greater level of personal strength.

The complete regression model significantly explained 12.3% of the variance of the scores of the PTG dimension of new appreciation of life (*R*^2^ = 0.123, *F* = 3.46, *p* < 0.001). The number of traumatic events one has experienced, sex, and posttraumatic stress symptoms were significantly associated with the dimension of new appreciation of life (partial *r* = 0.21, 0.17, and 0.13, respectively). This implies that having suffered more traumatic events after the terrorist attack, being a woman, and experiencing more posttraumatic stress symptoms, in this order, were associated with a greater level of a new appreciation of life.

Finally, the complete regression model significantly explained 7% of the variance in spiritual change scores (*R*^2^ = 0.070, *F* = 2.11, *p* = 0.044), but, once again, only sex and posttraumatic stress symptoms were significantly associated with spiritual change (partial *r* = 0.14 in both cases). These associations indicated that being a woman and a greater degree of posttraumatic stress symptoms were associated with greater PTG in spiritual change.

All these regression analysis results were not affected by collinearity issues, given that, in each analysis, all tolerance rates were higher than 0.52 and all VIFs were lower than 1.93.

### Variables Associated With Posttraumatic Growth: Curvilinear Relationship

Table 5 shows the results of the hierarchical regression analysis that were carried out to examine the possible curvilinear relationship between the PTG measures and continuous variables that were assessed in this study. These include sociodemographic and clinical variables, as well as variables related to the attack. The inclusion of the quadratic term of these variables (e.g., posttraumatic stress symptoms) did not imply a statistically significant increase in the percentage of explained variance of any of the PTG when contrasted with a model that initially only included a linear term of any of the given variable. For this reason, none of the predictors’ quadratic components were statistically significant, meaning that there were no significant curvilinear relationships between the PTG measures and sociodemographic, clinical, and terrorist attack-related variables that are shown in Table 5.

**TABLE 5:** Linear and curvilinear relationships between the continuous predictors and the posttraumatic growth measures from the PTGI: Multiple regression analyses.

Predictor	Posttraumatic growth measures from the PTGI											
	PTGI total score		Relating to others		New possibilities		Personal strength		Appreciation of life		Spiritual change	

	B	Δ*R*^2^	β	Δ*R*^2^	β	Δ*R*^2^	β	Δ*R*^2^	β	Δ*R*^2^	β	Δ*R*^2^
**Posttraumatic stress symptoms**												
Step 1: Linear term	0.100	0.010	0.109	0.012	0.077	0.006	−0.011	0.000	0.168*	0.028*	0.138*	0.019*
Step 2: Quadratic term	−0.048	0.001	−0.030	0.000	−0.012	0.000	−0.086	0.004	−0.043	0.001	−0.021	0.000
**Depressive symptoms**												
Step 1: Linear term	0.038	0.001	0.056	0.003	0.001	0.000	−0.047	0.002	0.117	0.014	0.104	0.011
Step 2: Quadratic term	−0.155	0.012	−0.093	0.004	−0.152	0.011	−0.105	0.005	−0.185	0.017	−0.171	0.014
**Years elapsed since the attack**												
Step 1: Linear term	−0.046	0.002	−0.041	0.002	−0.060	0.004	0.005	0.000	−0.108	0.012	0.003	0.000
Step 2: Quadratic term	−0.064	0.003	−0.056	0.002	−0.042	0.001	−0.062	0.003	−0.124	0.011	−0.018	0.000
**Optimism**												
Step 1: Linear term	0.044	0.002	−0.028	0.001	0.095	0.009	0.146*	0.021*	−0.010	0.000	−0.030	0.001
Step 2: Quadratic term	−0.126	0.014	−0.062	0.003	−0.135	0.016	−0.083	0.006	−0.186	0.030	−0.132	0.015
**No. of terrorist attacks**												
Step 1: Linear term	0.131	0.017	0.155*	0.024*	0.078	0.006	0.119	0.014	0.081	0.007	0.037	0.001
Step 2: Quadratic term	0.059	0.001	0.031	0.000	0.099	0.002	0.078	0.001	0.101	0.002	0.040	0.000
**No. of traumatic events after the attack**												
Step 1: Linear term	0.201*	0.041*	0.189*	0.036*	0.167*	0.028*	0.178*	0.032*	0.214*	0.046*	0.031	0.001
Step 2: Quadratic term	0.107	0.004	0.137	0.006	0.168	0.009	−0.082	0.002	0.078	0.002	0.248	0.019
**Age**												
Step 1: Linear term	0.104	0.011	0.086	0.007	0.066	0.004	0.163	0.026*	0.021	0.000	0.108	0.012
Step 2: Quadratic term	−0.013	0.000	−0.022	0.000	0.001	0.000	0.037	0.001	−0.117	0.014	0.043	0.002
**Education level**												
Step 1: Linear term	−0.060	0.004	−0.023	0.001	−0.034	0.001	−0.108	0.012	−0.055	0.003	−0.052	0.003
Step 2: Quadratic term	−0.116	0.013	−0.092	0.008	−0.088	0.007	−0.110	0.012	−0.131	0.016	−0.129	0.016
**Age at the time of the attack**												
Step 1: Linear term	0.105	0.011	0.092	0.008	0.067	0.004	0.135	0.018	0.078	0.006	0.078	0.006
Step 2: Quadratic term	−0.014	0.000	−0.016	0.000	0.010	0.000	−0.031	0.001	−0.074	0.005	0.064	0.004

*Statistically significant predictor at p < 0.05.

## Discussion

The main objective of this study was to examine the presence of long-term PTG in people who have been directly exposed to a terrorist attack. To our knowledge, there are only two published studies that have addressed a similar objective with a large enough sample to obtain solid results and conclusions. In addition, both studies were based on the same sample of survivors of the 1995 Oklahoma City terrorist attack (Tucker et al., 2016, 2018). The present study was conducted using a sample of people who had been directly exposed to a terrorist attack in Spain, 29.6 years ago on average. The results obtained in this study indicate that after suffering a terrorist attack, approximately 25–44% of people will show moderate or high levels of long-term PTG. In other words, even after so many years and even after having directly suffered such a devastating event as a terrorist attack, a significant amount of people will experience PTG.

However, this percentage is significantly smaller than that found in the meta-analysis conducted by Wu et al. (2019), where 53% of people who had been exposed to different types of traumatic events experienced moderate-to-high PTG. The difference between these percentages can be explained by the specific characteristics of the traumatic event assessed in the present study. In fact, Wu et al. (2019) found in their meta-analysis differences in the prevalence of PTG according to the type of traumatic event people had suffered. Terrorist attacks are considered more devastating than other disasters or types of violence for the following reasons: (a) they deliberately intend to cause harm to others; (b) they target large social groups or society itself; and (c) there is a constant threat without a clear ending, reason for which no one can be sure if the worst is yet to come (Vázquez et al., 2008). The difference could also be justified by the amount of time elapsed since the occurrence of the traumatic event. In the meta-analysis conducted by Wu et al. (2019), a shorter amount of time passed since the experience was associated with a higher prevalence of moderate-to-high PTG. However, when the results of the present study are compared to the ones obtained in the studies carried out by Tucker et al. (2016, 2018), those two explanations seem insufficient.

Tucker et al. (2016, 2018) also used a sample of people who had been directly exposed to terrorist attacks many years before being evaluated. The prevalence of moderate-to-high PTG was also significantly lower in the present study compared to the one conducted by Tucker et al. (2016). In fact, the mean level of PTG found in the victims of terrorism included in the present study was significantly lower than the one found amongst the survivors of the 1995 Oklahoma City terrorist attack (Tucker et al., 2018).

Taking this into consideration, another possible explanation for the statistically significant differences between samples has to do with the political, social, and cultural context in which the terrorist attacks occurred, as well as the characteristics of the terrorist attacks themselves. The terrorist attack that took place in Oklahoma City in 1995, was a single unrepeated episode and was not associated to a serious civil or political conflict. In addition, the terrorists did not belong to an organized terrorist group from their own social group or community. As suggested by Vázquez et al. (2008), terrorist attacks can have a greater personal and collective impact when they are perpetrated by organized terrorist groups that belong to the community, and when they occur in the context of a serious ongoing civil or political conflict. Examples of places where this kind of terrorism took place are Northern Ireland, Sri Lanka, or the Basque Country in Spain. According to Vázquez et al. (2008), this type of terrorism creates a collective atmosphere of wariness, mistrust, and destruction of the moral system of the community. It is important to emphasize that this type of terrorism also implies a high and continued threat level both to direct and indirect victims. Not only because of the continuity of the attacks, but because of the repetitive and prolonged acts of physical and psychological violence.

In Spain, ETA’s terrorist activity did not only consist in the assassination of specific people by shooting them in the nape or by placing explosives in their vehicles. ETA also intended to kill indiscriminately by placing powerful bombs close to households, shopping centers, or on the street. Although most of the targets were usually members of security forces, military, and their families, ETA also targeted politicians, judges, journalists, prosecutors, university professors, or businesspersons. To these fatal attacks, we must also add the frequent acts of street violence, where juvenile organizations related to ETA burnt or destroyed houses, businesses, bank offices, political headquarters, public busses, vehicles, etc. According to López Romo (2015), a total of 5,113 street violence incidents took place in the Basque Country between 1991 and 2013. In 2002, 963 people (politicians, judges, prosecutors, journalists, professors, etc.) had to be escorted for protection due to the ETA’s threats (without considering members of the armed and security forces, all of them targeted by ETA). Furthermore, an average of 804 terrorist attacks and street violence incidents took place per year between 1995 and 2000, and during the “years of lead” regarding terrorism in Spain (1978–1988), more than 65 people were assassinated on average per year. According to Martín-Peña (2013), victims of ETA and those who were threatened by the terrorist group in the Basque Country also suffered psychological violence: 69% were watched and controlled by people related to ETA, 74% received threats, 79% were disrespected, humiliated, or rejected, and 90% felt stigmatized. These data indicate that there are differences between the victims of terrorism in Spain and the victims from the 1995 Oklahoma City terrorist attack. The victims of terrorism in Spain were exposed for many years to repeated and intense terrorist attacks, as well as repeated physical and psychological acts of violence in a collective atmosphere of wariness, mistrust, and threat.

In this violent and threatening social context, it seems coherent that the percentage of people who had experimented large or very large changes was significantly smaller for the victims of terrorism that took part in the present study compared to the victims of the 1995 Oklahoma City bombing. Not only that, but they also obtained significantly lower scores in some items from three out of the five PTG dimensions: Personal Strength, Relating to Others, and Spiritual Change, especially in the two last dimensions. However, when explaining these findings, it is also necessary to address the cultural differences between Spain and the United States. For example, only 27% of the Spanish population consider that religion has an important and central role in their lives, compared to 62% of the United States population (Vázquez and Páez, 2010). This cultural difference could partially explain the smaller increase in spiritual growth amongst the sample of Spanish victims that took part in the present study. Along the same lines, various studies have found transcultural differences in PTG in response to different types of traumatic events (see Tedeschi et al., 2018). An example of this is the smaller degree of PTG in Spanish university students that were not directly exposed to the terrorist attacks of March 11, 2004, in Madrid, compared to university students from the United States that were not directly exposed to the terrorist attacks of September 11, 2001, in New York and Washington, DC (Steger et al., 2008). This last finding has been interpreted as a reflection of the differences between collectivistic and individualistic cultures. It is more probable for people that come from individualistic cultures to make a greater effort to strengthen positive self-views through self-enhancement (Steger et al., 2008). Coherently, the United States is considered a much more individualistic country compared to Spain (a mean score of 91 vs. 51 in the individualism/collectivism dimension proposed by Hofstede; Vázquez and Páez, 2010).

These cultural differences and their possible influence on long term PTG after a terrorist attack should be further researched. When doing so, certain variables such as sex must be controlled, given that in this study it was the most consistent and strongly associated variable with long-term PTG. In this sense, it is worth pointing out that the present study and the studies conducted by Tucker et al. (2016, 2018) had a similar percentage of women (49.5% vs. 52.2%) and a similar mean age (53.48 vs. 58.7 years). However, both samples differed in important characteristics, such as the type of exposure to the terrorist attack. The people who took part in the studies of Tucker et al. (2016, 2018) were all survivors, while the people who took part in the present study were not only survivors (38.6%). They were also close relatives of persons who had been wounded in a terrorist attack (20%) or close relatives of persons who had been killed in a terrorist attack (41.4%). The fact that a high percentage of people who took part in this study were close relatives of persons who had been killed in a terrorist attack could help explain the high levels in the PTG dimension of appreciation of life when compared to those obtained in the studies of Tucker et al. (2016, 2018). This data is consistent with that obtained in previous studies. Compared to survivors of other types of traumatic events, such as victims of sexual assault, people who suffered the loss of a family member report higher levels of PTG, specifically in the dimension of appreciation of life (Shakespeare-Finch and Armstrong, 2010).

The present study also intended to examine the variables that could influence long-term PTG in people directly exposed to terrorist attacks. Out of the 16 sociodemographic, clinical, and attack-related variables that were gathered in this study, sex was the variable that showed a greater association with long-term PTG. Sex was also the only variable that was consistently associated with all the PTG measures, with coefficients between small and moderate (range of *r* = 0.17–0.29), reflecting that women reported greater levels of PTG than men.

This finding is coherent with the results obtained in the meta-analysis conducted by Vishnevsky et al. (2010) that included multiple types of traumatic events and came to show a difference in PTG between sexes in the same direction and between small and moderate (*g* = 0.27, equivalent to *r* = 0.134). It is also consistent with the results obtained by Tucker et al. (2016) on long-term PTG in terrorist attack survivors. For these reasons, the results obtained in the present study prove the importance of the sex of the person when explaining individual differences in PTG, even long-term and after suffering a terrorist attack. The results also show a need for further investigation on the specific factors that differentiate men from women and that could mediate the differences in PTG. For example, the sex differences in reflective or deliberate rumination processes, or emotion-focused coping, could be good candidates to be those mediators (Vishnevsky et al., 2010).

In this study, the second variable that was significantly and consistently associated with the long-term PTG measures was the number of traumatic events a person has suffered after the terrorist attack. The association of this variable with the total score of the PTG and with four of its five dimensions was positive and linear, but not curvilinear. This finding is coherent with the results obtained in previous studies that indicate that experiencing different types of traumatic events (cumulative trauma) is positively associated with global PTG and with several of its dimensions, such as a new appreciation of life, personal strength, and new possibilities (Kira et al., 2013). These results reaffirm the need to analyze in a more detailed manner the profile and characteristics of the traumatic events that a person has suffered in order to understand the presence of PTG.

In this study, the third variable that was significantly and consistently associated with the long-term PTG measures was posttraumatic stress symptoms. This association was positive and linear, but not curvilinear, with two PTG dimensions: New Possibilities (*r* = 0.16) and Spiritual Change (*r* = 0.13). This finding is consistent with the results of the meta-analysis conducted by Liu et al. (2017) on PTG after different types of traumatic events, although the mean correlation obtained in the meta-analysis was slightly superior to the ones obtained in the present study (0.22 vs. 0.16–0.13). The results of the present study are also coherent with the ones found in Tucker et al. (2016), where the association between long-term PTG and posttraumatic stress symptoms in victims of terrorism was significant and positive. In fact, the present study broadens the results obtained by Tucker et al. (2016), given that they did not examine the possibility of a curvilinear association in the shape of an inverted U (quadratic association) between long-term PTG and posttraumatic stress symptoms. The present study examined the presence of a curvilinear association for both the global PTG score as well as its five dimensions, but the results did not reveal statistically significant quadratic associations.

The absence of a curvilinear association between PTG and posttraumatic stress symptoms is not consistent with the results obtained from the meta-analysis of Shakespeare-Finch and Lurie-Beck (2014). These researchers found that the quadratic association was significant and in fact stronger than the linear association. However, the authors also found that the strength and linearity of the associations were different depending on the type of traumatic event that people had been exposed to. In addition, although Shakespeare-Finch and Lurie-Beck (2014) acknowledged the importance of elapsed time since the traumatic event, they were not able to examine its influence in the associations. For these reasons, it is possible that the absence of a quadratic association between PTG and posttraumatic stress symptoms is specific to long-term PTG after suffering a terrorist attack, as the results of the present study indicate.

In sum, there is a positive association between posttraumatic stress symptoms and long-term PTG in people directly exposed to a terrorist attack. In particular, there is a positive association of posttraumatic stress symptoms with the PTG dimensions of appreciation of life and spiritual change. This supports the idea that a certain degree of emotional distress is a necessary condition to develop PTG (Tedeschi et al., 2018). According to the results obtained in the present research, that emotional distress derived from the traumatic event would not be related to depressive symptoms, but to posttraumatic stress symptoms. In addition, the positive association between posttraumatic stress symptoms and long-term PTG suggests that positive psychological changes are not sufficient to eliminate the suffering caused by the direct exposure to a terrorist attack.

Finally, the results of the present study indicate significant, positive, and linear associations between the PTG dimension of relating to others and the number of terrorist attacks suffered by the victim. Also, between the PTG dimension of personal strength and the victim’s age. However, in this study, no linear or quadratic associations were found between long-term PTG or its dimensions and other sociodemographic, clinical, and attack-related variables. In particular, they were not found between long-term PTG and depressive symptoms, optimism, education level, marital status, the diagnosis of an emotional disorder (PTSD, MDD, or anxiety disorder), the age the person had when he/she suffered the attack, the type of exposure to the attack (injured, family member of someone deceased or injured) and the time elapsed since the terrorist attack. Regarding this last variable, the present study examined a time span of 2 to 47 years since the terrorist attack, while the meta-analysis conducted by Wu et al. (2019) examined a time span of a few days or months to a few years after the terrorist attack. Given that Wu et al. (2019) found a significant and negative association between the time elapsed since a traumatic event and PTG, it is possible that this association could disappear after many years. In particular, there is a possibility that this might happen with terrorist attacks of similar characteristics as the ones suffered by the victims of the present study.

The absence of a significant association between long-term PTG and depressive symptoms is consistent with the findings of Tucker et al. (2016) in a sample of survivors of the 1995 Oklahoma City bombing. It is also consistent with results of the meta-analysis conducted by Long et al. (2021) on different types of traumatic events. However, the absence of a significant association between long-term PTG and optimism is not consistent with the results obtained in the meta-analysis conducted by Prati and Pietrantoni (2009). These authors found an almost moderate association (*r* = 0.23) between PTG and optimism in people who had experienced all sorts of traumatic events. Nevertheless, they did not examine the moderating role of the type of traumatic events that victims had suffered on the association between PTG and optimism. The association between long-term PTG and optimism was also not examined in the studies conducted by Tucker et al. (2016) on survivors of the 1995 Oklahoma City terrorist attack. For these reasons, once other variables such as sex or posttraumatic stress symptoms are controlled, it could be possible to find that there is no association between optimism and long-term PTG in victims of terrorist attacks.

This possibility and the fact that there is not much literature that addresses this topic emphasize the need to carry out new studies on long-term PTG in people who have been directly affected by a terrorist attack. Future studies must try to surpass the limitation of the present study, which should be taken into consideration when considering its results and conclusions. Amongst these limitations, we would like to point out that this study has a cross-sectional design. For this reason, it would be necessary to carry out longitudinal studies, allowing a better evaluation of PTG evolution across the years and assessing the influence of different factors throughout this evolution. Another limitation has to do with the response rate of the study and, therefore, with the representativeness of the sample and the generalization of the results obtained by said sample. The response rate in the present study was between 26.5% (regarding the people who had been contacted in the first phase of the study) and 49.1% (regarding the people who had been contacted in the second phase). These data are consistent with those obtained in other studies conducted with people who had been directly affected by terrorist attacks in Spain (e.g., Miguel-Tobal et al., 2006). In the present study, no statistically significant differences were found between the people who took part in the face-to-face interview and those who did not when considering their marital status, education level, sex, or the time elapsed since the terrorist attack. However, there were statistically significant differences regarding the age of the person at the time of the evaluation, the age of the person when the attack took place, the type of exposure to the attack, and the presence of emotional symptoms. For this reason, a certain selection bias linked to the response rate cannot be completely ruled out.

Another limitation that should be acknowledged has to do with the possibility of correctly assessing PTG many years after the exposure to the terrorist attack. This limitation is related to a more general matter, the validity of reports of PTG. This matter has been thoroughly debated through the scientific literature and is faced with various difficulties (Tedeschi et al., 2018). Of course, these difficulties are even greater when PTG is assessed long-term. Some of these difficulties have to do with the idea that what PTG instruments such as the PTGI really assess is self-reported or perceived PTG, and not necessarily “authentic” or actual PTG. In this sense, self-reported or perceived PTG could be subject to the biases that the person being evaluated could have, including self-serving biases. In the case of long-term PTG, we should also take into consideration memory biases that could take place due to the elapsed time, and that could affect the reliability of self-report data. Taking this into account, future investigations should explore the degree of agreement between self-reported PTG and the changes in PTG observed by the person’s family members, friends, or co-workers. An additional problem when assessing long-term PTG is related to the difficulty to discriminate if the changes in one’s perception of him or herself, the world, and life are linked to the traumatic event he/she experienced or to the passage of time, for example, moving on from a young age to middle age or from middle age to old age. For this reason, future studies should include a control group to ensure that the evaluated growth has really emerged from the traumatic event and not only from the passage of time.

Despite its limitations, the results obtained in present study reveal new information on long-term PTG in people who have been directly affected by a terrorist attack, and there are hardly any studies on this type of PTG in this specific population. These results emphasize the influence of sex and cumulative trauma on long-term PTG and support the idea that a certain degree of posttraumatic stress symptoms is a necessary condition to develop PTG. The results also come to show the need for further research regarding long-term PTG, taking into consideration the characteristics of the terrorist attack and the profile and characteristics of other traumatic events suffered after the attack. It is also important to consider the context of violence and threats to which the victims are or were exposed, as well as the political, social and cultural characteristics of the communities affected by the attacks.

## Data Availability Statement

The raw data supporting the conclusions of this article will be made available by the authors upon reasonable request.

## Ethics Statement

The studies involving human participants were reviewed and approved by Ethics Committee of the Faculty of Psychology at Complutense University of Madrid (Spain). The patients/participants provided their written informed consent to participate in this study.

## Author Contributions

RF, JS, and MG-V contributed to conception and design of the study and wrote sections of the manuscript. RF, AN-M, CG, NM, BC-R, PA, JM, and AS-G collected the data and organized the database. JS and RF performed the statistical analysis and wrote the first draft of the manuscript. All authors contributed to manuscript revision, read, and approved the submitted version.

## Conflict of Interest

The authors declare that the research was conducted in the absence of any commercial or financial relationships that could be construed as a potential conflict of interest.

## Publisher’s Note

All claims expressed in this article are solely those of the authors and do not necessarily represent those of their affiliated organizations, or those of the publisher, the editors and the reviewers. Any product that may be evaluated in this article, or claim that may be made by its manufacturer, is not guaranteed or endorsed by the publisher.

## Footnotes

1 Sanz, J., Altungy, P., Reguera, B., Navarro, R., Gesteira, C., Sanz-García, A., et al. (2021). *Psychometric Validation of the Spanish Version of the Posttraumatic Stress Disorder Checklist for DSM-5 (PCL-5)*. Under review.
